# Reasons for delay in seeking treatment among women with obstetric fistula in Tanzania: a qualitative study

**DOI:** 10.1186/s12905-019-0799-x

**Published:** 2019-07-10

**Authors:** Mary A. Lyimo, Idda H. Mosha

**Affiliations:** 1 School of Nursing and Midwifery, The Aga Khan University, PO Box 13129, Dar es Salaam, Tanzania; 2 Behavioural Sciences Department, School of Public Health and Social Sciences, Muhimbili University of Health & Allied Sciences, PO Box 65004, Dar es Salaam, Tanzania

**Keywords:** Delay in seeking treatment, Obstetric fistula, Distance, Knowledge, Social culture

## Abstract

**Background:**

Obstetric fistula is among the serious and distressing maternal morbidities in Tanzania. Obstetric fistula is a childbirth-related injury caused by prolonged and obstructed labor which has a devastating impact on affected women and their families. The aim of this study was to explore reasons why women with obstetric fistula admitted to the Comprehensive Community- Based Rehabilitation in Tanzania (CCBRT) hospital delayed seeking fistula treatment.

**Methods:**

This exploratory study incorporated qualitative approach. In-depth interviews were used to collect data from 18 women with obstetric fistula admitted to CCBRT hospital. The interviews were conducted in Kiswahili and lasted for 40–45 min. Audio-recordings of the interviews were transcribed verbatim and translated into English. Thematic analysis was used to extract reasons for the delay in seeking treatment for obstetric fistula.

**Results:**

The study sample (*n* = 18) ranged in age from 20 to 57 (μ = 37; SD = 11.67), married (*n* = 14), unemployed (*n* = 15), and very low level education (*n* = 15) with primary education. Delay in seeking treatment for obstetric fistula was related to the following themes: *inadequate knowledge about the causes and treatment of obstetric fistula, distance and transport cost to a health facility, stigma, community isolation, social isolation and use of traditional and cultural.*

**Conclusion:**

The Tanzanian Ministry of Health in collaboration with private institutions should strengthen education programmes on the nature and causes of obstetric fistula, and increase the availability of treatment to decrease the effect of this condition for women in Tanzania.

**Electronic supplementary material:**

The online version of this article (10.1186/s12905-019-0799-x) contains supplementary material, which is available to authorized users.

## Background

Obstetric fistula refers to a hole between the vaginal wall and bladder or rectum resulting from lack of blood supply/flow. This causes the tissue to die, leaving affected women incontinent of faeces or urine or both. Obstetric fistula is caused by obstructed and prolonged labor [1, 2]. Globally, 8% of maternal death is caused by obstetric fistula, and girls and women with untreated obstetric fistula experience isolation, shame, and poverty [3]. Currently, an estimated 2–3.5 million girls and women are living with obstetric fistula [4], and 50,000–100,000 cases of fistula occur each year worldwide [5, 6]. In Tanzania, more than 21,400 women are living with untreated obstructed fistula [7], and there are around 2,500–3,000 new cases each [1, 8]. However, no population-based surveys on obstetric fistula have been undertaken, and data remain [9].

A study conducted in Tanzania and Uganda on the childbirth experiences of women with obstetric fistula revealed that 53% of these women had waited for more than a year before seeking medical treatment [10]. The Tanzanian government has attempted to identify all girls and women with untreated fistula and refer them to healthcare facilities for free surgical repair [11]. Repair of fistula is recommended within 3 months of woman first experiencing fistula symptoms; if women delay seeking treatment for more than 3 months, the condition tends to be difficult to repair and results in more delay in healing and possible reoccurrence of fistula [12, 13]. Surgical fistula repair has a success rate of up to 90% and has been shown to have a positive impact on women’s lives by improving their economic status, physical condition and interpersonal relationships [12].

Despite prevention efforts and free treatment of obstetric fistula, many women in the community delay seeking treatment and continue to live with fistula [14]. The reasons why women with obstetric fistula in Tanzania delay seeking medical help are not well known. To our knowledge, no studies have investigated factors contributing to the delay in seeking obstetric fistula treatment in Tanzania. Therefore, this study explored reasons for the delay in seeking medical treatment among women with obstetric fistula admitted to the Comprehensive Community-Based Rehabilitation in Tanzania (CCBRT) hospital.

## Methods

### Study design and setting

Exploratory qualitative study using in-depth interviews (IDI) was used to explore reasons for the delay in seeking treatment among women with obstetric fistula admitted to CCBRT hospital in Tanzania [15]. Data was collected through in-depth interviews based on an interview guide. This study was conducted from May to June 2018 in Dar es Salaam, Tanzania.

### Sampling method

Purposeful sampling was used to select the study participant. The patients with obstetric fistula admitted at CCBRT hospital were selected and included in the study after consenting. Purposive sampling allowed the researcher to select the sample that could facilitate rich description and understanding of the phenomena under investigation based on the study topic [16]. In order to get the intended participants, the meeting between the researcher and nurse in-charge of the CCBRT fistula ward was held prior to data collection (see Additional file 1). In the meeting, we discussed about the aims of the study, inclusion (All women with obstetric fistula admitted to CCBRT hospital who had delayed seeking treatment for more than 3 months since the onset of symptoms) [17], exclusion criteria and ethical consideration of the study.

The researcher intended to recruit 20 study participants. However, enrolments of 18 participants were determined by the principle of data saturation where the responses does not give any new information and the interview stopped [16, 18], and all 18 participants completed all interview questions and none of them refused or drawn from the study.

### Data collection

An interview guide was used to collect information from participants through in-depth face to face interviews. The interview guide was developed by reviewing different literature and consulting an expert [19]. The interview guide covered demographic characteristics, sociocultural factors, knowledge and physical factors contributing to delay seeking fistula treatment. The in-depth interview (IDI) guide was developed in English and translated into Kiswahili by the first author. The interviews were conducted at CCBRT hospital, Tanzania in Kiswahili (Tanzania’s national language), which is universally accessible and understood by the majority of Tanzanians. The first author establishes relationship with staff and participants prior to the interviews [18]. Each interview lasted 40–45 min.

### Data analysis

Data were transcribed and analyzed using thematic analysis. Thematic analysis using five stages according to Braun and Clarke was performed to establish meaningful patterns: familiarisation with the data, generating initial codes, searching for themes among codes, reviewing themes and presenting the results [20, 21]. Nvivo 12 version computer software was used to aid data analysis process. Table 1 Illustration of the data analysis of the text shows how the data were analyzed.

**Table 1:** Illustration of the process of data analysis

Text	Codes	Sub theme	Theme
*The fistula condition is irritable. You find yourself wet all the time*. *.*. *and isolating yourself from your fellows. Imagine they bring food; you share with them while every time you touch urines. Even if you wash your hands, still I don’t feel good. It is like you are causing discomfort to them. (IDI, 42 years)*	Fear of staying with others	Afraid being stigmatized Self-isolation	Stigma against women with fistula condition
	Separate yourself from others Discomfort		
*First of all I was maltreated and abused. They isolated me, stigmatized me, my husband disowned me, my mother-in-law refused me, and they said I was stinking*. *.*. *I suffered so much. On my way to hospital, someone said, “The one, who urinated in the bus yesterday, will be dropped on the way.” Another person said, "No, she didn’t urinate purposefully in the bus. (IDI, 18 years)*	Maltreated and abused	Being isolated by the others Community isolation	
	Separated by others		
	Rejected by spouse		
	Bad words Threatening		

## Results

### Participants’ background characteristics

The socio-demographic characteristics of the 18 participating women are shown in Table 2. The results shows that majority of participants were married (*n* = 14), unemployed (*n* = 15), with primary education (*n* = 15) with low level of education, and the mean age of respondent are 37 years with standard deviation of 11.67.

**Table 2:** Participants’ demographic data

Marital status	Marital status	Frequency	Percent
	Divorced	2	11.11
	Married	14	77.78
	Widow	2	11.11
	Total	18	100
Occupation	Occupation	Frequency	percent
	Self- employment	3	16.67
	Unemployed	15	83.33
	Total	18	100
Level of education	Level of education	Frequency	Percent
	None	2	11.11
	Primary	15	83.33
	Secondary	1	5.56
	Total	18	100
Respondents age	Statistics	Age	
	N	18	
	Mean	37.22	
	Std. Deviation	11.67	

The main themes associated with the delay in seeking obstetric fistula treatment reflected socio-cultural factors (*traditional practice and Stigma against women with obstetric fistula*), poor knowledge about obstetric fistula *(women and community knowledge on obstetric fistula)* and transport/distance issues in seeking fistula treatment.

### Socio-cultural factors

Tanzania has many tribes with different beliefs and cultural practices that tend to either directly and indirectly affects the health of a woman. In Tanzania, decision making in all matters at a family and community level is predominantly made by men. This has contributed to unequal opportunities to education and lack decision on when and where to seek healthcare services. Majority of Tanzania’s believes that disease occurrence may have been influenced by witches or punishment from God hence concentrate more on seeking spiritual healing from religious leaders and spiritualist (*witchdoctors)*. Hence women choices about their reproductive health get affected resulting in delayed women in seeking early fistula treatment in Tanzania [22]. This theme is divided into sub-themes which are traditional practice and stigma against women with obstetric fistula as described below.

#### Traditional practice

Most participants had tried traditional practices (spiritual therapies and traditional herbs) when seeking obstetric fistula treatment. Traditional practice is highly influenced through word-of-mouth, and many women with an obstetric fistula sought a traditional healer (witch doctor) for treatment as a result of being influenced by relatives, their spouse, and community. Participants believed traditional healers were their first option for help with their health problems. One participant commented that:

*Others said people have bewitched me. They said it was caused by witches. But I said no, it wasn’t that. Some people said, ‘We told you not to go to the hospital. Now, do you see what they did to you? If you remained here in the village you wouldn’t get this disease’. So, some people were laughing at me for going to the hospital. (IDI, 52 years).*

Many participants reported that relatives and community members recommended they seek treatment from local healers because of a belief that the woman’s condition was associated with witchcraft; therefore, going to a healthcare facility for medical attention would be wasting time and money. Participating women also consumed traditional herbs and performed religious observances with the belief that their condition would be healed through praying and the use of traditional medicine recommended by local traditional healers. An example of such a view is presented below.

*People say that we have to seek traditional medicine because it is not a mere fistula condition; they say that fistula is caused by being bewitched. Therefore, I have gone to so many witch doctors trying to get treatment and use traditional herbs in vain. (IDI, 35 years).*

Another reason for the delay in seeking fistula treatment was a belief that fistula was caused by their ancestors’ curse. These women took a lot of time to seek appropriate clan/traditional elders who could assist them to find out the cause of the curse and solution to their problem. Advice to seek a cure for a curse from ancestors was given by relatives and friends. One woman commented that:

*Relatives and friends asked me to go to the elders to find out whether our ancestors have caused that. But I said how can ancestors bring me this malady? But I didn’t go to any traditional doctors. Even my mother told me to do so, citing that it may be the ancestors of our clan that has brought me this problem. I said if hospital treatment would be available, I will go there. (IDI, 51 years).*

Participants revealed that the community at large played a role in them delaying seeking treatment in healthcare facilities. Community members around women with obstetric fistula would advise and urge these women to seek local healers, which resulted in prolonged conditions and increased pain. One participant shared that:

*Neighbours and residents are told to go to witchdoctors first, they go and rituals are performed, promised that they will be healed. But when they return home, they find that the condition is the same. No healing. In our Maasai tribe, they believe that fistula is caused by witchcraft. (IDI, 30 years).*

#### Stigma against women with obstetric fistula

Stigma was another major factor that delayed women seeking obstetric fistula treatment. Stigma was often described as self-isolation whereby women with fistula isolated themselves from (and were isolated by) their loved ones and community occasions. Participating women reported isolating themselves because of their condition (e.g. incontinence, shame, and bad smells). In turn, this increased their fear of disclosing their condition, which contributed to lack of information about the condition, available treatment and where to get treatment, and culminated in further delay in seeking fistula treatment.

#### Self-isolation

Self-stigma resulted in women’s self-isolation from their loved ones and communities. Participating women reported they isolated themselves because of wet clothing and bad smells, which made them, feel too shy to sit near others and participate in community events. Self-stigma negatively affects self-esteem and self-efficacy and triggers the feeling of guilt and shame, which may lead women to avoid seeking fistula care. In this context, self-isolation contributed to the delay in seeking fistula treatment because women failed to disclose their condition and seek information concerning fistula treatment.

On participant reported that:

*If I see my clothes are wet wherever I would be sitting, I would get up quickly, run to the cassava farm and dry them in the sun. And even the bed that I was using I would first put a waterproof material and then I would be the first to get up in the morning, run to the field and put my bed sheets up to dry and make sure nobody sees me and knows about my condition. (IDI, 45 years).*

#### Community isolation

Public stigma in terms of isolation by community members/neighbors and loved ones (e.g. family members or spouses) was commonly reported by participants. Community/family members isolated women with obstetric fistula from participating in different events/occasions, and their loved ones isolated them by actions such as not sharing domestic tools. Some women ended up being divorced because they were suffering from obstetric fistula. Such stigma may lead individuals to avoid seeking medical help, as they expect others to discriminate against them. They may also fear negative reactions from others because of bad smells and wetness, and adopt behaviors such as hiding themselves from others, not sharing anything about their condition and failing to seek help or visit a hospital. The effect of this isolation and stigma was clearly described by one participant:

*First of all, I was maltreated and abused by people. People isolated me, stigmatized me, my husband disowned me, my mother-in-law refused me, and they said I was stinking. I suffered so much and sometimes found [I was] isolating myself from them and I don’t want to talk or go anywhere. (IDI, 18 years).*

### Knowledge about obstetric fistula

This study revealed a range of views among participating women with regard to the role of knowledge about obstetric fistula influencing the delay in seeking fistula treatment. Participants’ reports showed that lack of knowledge about fistula, mode of treatment, complications, and availability of treatment limited access to fistula treatment and resulted in delays in seeking treatment. The theme is divided into sub-themes: *Women’s knowledge of obstetric fistula and community knowledge about obstetric fistula* as explained below.

#### Women’s knowledge of obstetric fistula

Many participants had no knowledge about the meaning, cause, effects and treatment of obstetric fistula. Some reported that they understood the causes and effects of their condition after they had arrived at the CCBRT hospital for treatment, where they received daily classes on obstetric fistula. The lack of information about fistula treatment was cited by women as a reason for living with untreated fistula for a long time before seeking treatment. Lack of information about obstetric fistula meant that women failed to make decisions about seeking fistula treatment, which delayed treatment. Lack of information about fistula appeared to be related to participants’ level of education, as most women with low level of education delayed seeking fistula treatment.

The following excerpt from a study participant demonstrates the role of knowledge in seeking treatment.

*I didn’t know what was happening to me…I didn’t know what were the causes and treatment of this condition. The fistula condition was known to me after arriving here at the hospital, but where I come from, my neighbour told me fistula condition is treatable, and I just came here for treatment of urine outflow. (IDI, 45 years).*

#### Community knowledge about obstetric fistula

This study also found that family and community awareness about obstetric fistula was low. Most family members (relatives and spouses) were not aware of obstetric fistula as reported by fistula patient, which contributed to the delay in women accessing treatment centres and resulted in more negative effects. Some women with obstetric fistula were separated from their spouses after developing obstetric fistula, and some were stigmatized by their community and relatives. Women with fistula reported that their families played a major role in their delay in seeking fistula treatment. Lack of information about fistula treatment in the community meant that families did not give social and financial support to women with fistula, which further resulted in women seeking traditional treatment rather than medical care. This lack of obstetric fistula awareness at the community level was described on one participant:

*After delivering my child at the hospital, I suffered from fistula, I went back to my husband, I was mistreated by that family and say I will never be healed again, and they also advised my husband to marry another wife because of my condition. (IDI, 28 years).*

### Distance-related delay in seeking fistula treatment

Distance from healthcare facilities was a factor mentioned by some participants as playing a role in the delay in seeking fistula treatment. However, participants’ views on distance as a reason for the delay in seeking treatment varied by their location and age. Some participants spoke about barriers such as lack of transport, unfamiliar environment and lack of information. In contrast, other participants said that distance was not a problem as they could easily get transport and reach a health facility within a relatively short time.

*Transport is not really a challenge, because there are motorcycles from our home village to Ilula Township and from Ilula we go to Iringa Town by buses. I don’t know how many kilometres are there. But I think I can reach the hospital easily within a few hours. (IDI, 22 years).*

Another participant reported that lack of money was a reason for her delay in seeking fistula treatment.

*No, I have not been treated by traditional doctors. The problem was money, my relatives don’t have money … they didn’t have funds to send me there. So when I got this financial support from donors (CCBRT hospital) even my relatives rejoiced. (IDI, 50 years).*

The means of transport used by the majority of participants were motorcycles and buses from their home to the treatment center. Some participants used a tractor to reach a nearby main road to access other means of transport. This was because most participants were from rural areas, and needed to reach an urban area for treatment. However, their condition meant that they risked being isolated by people on buses because of wetness and bad smells, based on the belief that they could contaminate the buses and other passengers. This resulted in women continuing to live with untreated fistula. One participant shared that:

*Oh, I left my home by motorcycle, when the nurse phoned me I told her that the roads are bad and cars don’t come to this area regularly. She advised me to take a motorcycle to Matai, and board a bus from there. So I took a motorcycle, and then boarded a minibus to Sumbawanga town as it was not easy because they wanted to drop/leave me on the way due to wetness and bad smell. (IDI, 50 years).*

## Discussion

### Socio-cultural factors associated with seeking fistula treatment

This study revealed that families and communities around women with obstetric fistula had different understandings about the causes and treatment of obstetric fistula. Most participants thought that fistula was caused by being bewitched or cursed by ancestors. Such beliefs affected the behaviour of women seeking obstetric fistula treatment and often meant that they sought traditional medicines (mineral- or plant-based medicines and spiritual therapies). Similar results were noted by Kavai et al. [23], who reported that cultural beliefs and traditional practices reduced the use of medical care by women with obstetric fistula. That study also discovered that most women with obstetric fistula found it difficult to seek modern treatment because of their beliefs and cultural practices.

The findings of this study revealed that stigma was a major factor that prevented women from seeking timely obstetric fistula treatment. This was consistent with Khisa and Nyamongo [24, 25], who reported that women with fistula failed to reveal their condition because of stigma by community members. Women with fistula feared to lose their societal status in their community and found it difficult to speak about their problem for fear of the hatred they would receive. Fear and hatred influenced the delay in seeking fistula treatment because women were not willing to reveal their condition to others when they feared being stigmatized and isolated from the community.

### Knowledge about obstetric fistula

This study revealed that low knowledge was a major factor that hindered early seeking of fistula treatment. Participants reported inadequate knowledge about the meaning, causes, effects, and treatment of obstetric fistula. This was consistent with a study conducted in Ethiopia by Woldeamanuel [26] that showed most women with obstetric fistula lacked knowledge about the causes, treatment, and complications of their condition, and ascribed the condition to a curse or witchcraft.

Knowledge about fistula treatment may be inadequate in most rural communities in Tanzania; health information about fistula treatment in rural communities is urgently needed. This is supported by a previous study done by Tinuola & Okau [27], that reported health education, especially on reproductive health issues, was highly needed. Health education should be provided to women of reproductive age in rural areas where there is low knowledge about conditions such as obstetric fistula. Changing the culture that opposes timely access to treatment could be supported by creating a high level of awareness about obstetric fistula.

The inadequate knowledge about health problems related to obstetric fistula among communities and families revealed in this study may be compounded by the fact that prevention of communicable diseases in rural areas is performed by health workers, who tend to forget about issues such as obstetric fistula. The government of Tanzania in collaboration with non-governmental organizations (e.g., CCBRT), have made efforts to identify hidden fistula cases and disseminate health messages on causes, signs/symptoms, and treatment [11]. However, many women with obstetric fistula seek traditional treatment and delay seeking modern fistula treatment. Healthcare workers and all organizations working with community awareness programmes could review and re-design communications to improve knowledge about and reduce the burden of fistula.

### Distance in seeking fistula treatment

Women delayed seeking fistula treatment because of long distances, lack of transport and the cost of reaching treatment centers. This was consistent with a study conducted in Tanzania by Mselle and Kohi [28] which reported that distance and traveling cost to fistula treatment centres resulted in many women failing to access health facilities and living with untreated fistula. Inaccessibility or unaffordability of public transportation to reach a healthcare facility for fistula treatment caused women to delay seeking treatment. Many women used motorcycles or walked long distances, and some used other means to reach treatment centers. A study by Bangser [10] showed similar results, with transportation and cost being factors delaying women in seeking fistula treatment. That study highlighted that the majority of women with fistula were from villages, whereas most fistula treatment centers were in town centers. Furthermore, Mselle and Kohi [28] reported that women in some villages were unable to meet transportation costs. Therefore, many women delayed seeking obstetric fistula treatment and stayed at home despite the consequences and complications they might experience.

Poverty, limited education, and women making choice about their reproductive health are the cross-cutting issues which delayed women in seeking fistula treatment early in Tanzania [29] This study shows that most of the women are unemployed and having low level of education this affect their income and directly by delay seeking treatment due to lack of money for travelling to treatment center, treatment expenses and awareness of the disease itself. A married woman’s limited power in her household hinders her action for making care-seeking decisions; the shame associated with obstetric fistula is debilitating and often only overcome if a family member mitigates it with financial, psychosocial, and social support. The dynamics of women’s action and empowerment against (and within) socio-cultural norms of “leaking women” and implications about their sexuality, role in society, and surgical care seeking may vary by education status.

### Study limitations

The first author is a nurse midwife, and there is a possibility that the information provided by the participants was influenced by the researcher’s views about fistula. However, this was mitigated by concentrating on study objectives and the researcher was trained on qualitative methods which assisted in conducting the interviews. This study included participants who were able to travel to Dar es Salaam for treatment at CCBRT, something which excluded patients who could not travel to Dar es Salaam for treatment. This study looked at few factors which contribute to delay in seeking fistula treatment and left other factors which have been studied by other authors. Limited resources (e.g. time and funding) limited the present exploration at CCBRT hospital in Dar es Salaam Tanzania.

## Conclusion

Delay in seeking treatment for obstetric fistula remains a major problem in Tanzania. Our data suggest that some obstetric fistula victims bear a burden of stigma and social isolation because of lack of information on obstetric fistula. The *Ministry of Health*, Community Development, Gender, Elderly and Children (MoHCDEC) *Tanzania*. In collaboration with private institutions should strengthen education programmes on the nature and causes of obstetric fistula, and availability of treatment to decrease the incidences and negative effects of obstetric fistula among women in Tanzania.

## Additional file

Additional file 1: Patients selection process flow chart. (DOCX 26 kb)

## Data Availability

Data can be made available on reasonable request from the corresponding author.

## Abbreviations

CCBRT: Comprehensive Community Based Rehabilitation in Tanzania
UNAPF: United National Population Fund
WHO: World health organization

## References

[1] Mselle LT, Kohi TW (2015). Living with constant leaking of urine and odour: thematic analysis of socio-cultural experiences of women affected by obstetric fistula in rural Tanzania. BMC Womens Health.

[2] Organization WH (2006). Obstetric fistula: guiding principles for clinical management and program development.

[3] WHO Obstetric Fistula Report. WHO Traditional medicine strategy 2014–2023. 2015. http://www.who.int/traditional-complementary-integrative-medicine/publications/trm_strategy14_23/en/. Accessed 2 Aug 2018.

[4] Ahmed S, Tunçalp Ö (2015). Burden of obstetric fistula: from measurement to action. Lancet Glob Health.

[5] Polan Mary Lake, Sleemi Ambereen, Bedane Mulu Muleta, Lozo Svjetlana, Morgan Mark A. (2015). Obstetric Fistula. Disease Control Priorities, Third Edition (Volume 1): Essential Surgery.

[6] WHO. Annual technical report: 2013: Department of reproductive health and research, including UNDP/UNFPA/WHO/World Bank Special Programme of research training in human reproduction (HRP). Geneva: World Health Organization; 2014.

[7] CCBRT (2017). Annual Report.

[8] Cichowitz C, Watt MH, Mchome B, Masenga GG (2018). Delays contributing to the development and repair of obstetric fistula in northern Tanzania. Int Urogynecol J.

[9] Kimani ZM, Ogutu O, Kibe A (2014). The prevalence and impact of obstetric fistula on women of kaptembwa-Nakuru, Kenya. Int J Appl.

[10] Bangser M, Mehta M, Singer J, Daly C, Kamugumya C, Mwangomale A (2011). Childbirth experiences of women with obstetric fistula in Tanzania and Uganda and their implications for fistula program development. Int Urogynecol J.

[11] Fiander A, Ndahani C, Mmuya K, Vanneste T (2013). Results from 2011 for the transportMYpatient program for overcoming transport costs among women seeking treatment for obstetric fistula in Tanzania. Int J Gynecol Obstet.

[12] Dennis AC, Wilson SM, Mosha MV, Masenga GG, Sikkema KJ, Terroso KE (2016). Experiences of social support among women presenting for obstetric fistula repair surgery in Tanzania. Int J Women's Health.

[13] Mohamed AA, Ilesanmi AO, Dairo MD (2018). The experience of women with Obstetric Fistula following corrective surgery: a qualitative study in Benadir and Mudug regions, Somalia. Obstet Gynecol Int.

[14] Egziabher TG, Eugene N, Ben K, Fredrick K (2015). Obstetric fistula management and predictors of successful closure among women attending a public tertiary hospital in Rwanda: a retrospective review of records. BMC Res Notes.

[15] Creswell JW (2018). Qualitative inquiry and research design: choosing among five approaches.

[16] Palinkas LA, Horwitz SM, Green CA, Wisdom JP, Duan N, Hoagwood K (2015). Purposeful sampling for qualitative data collection and analysis in mixed method implementation research. Adm Policy Ment Health Ment Health Serv Res.

[17] De Bernis L (2007). Obstetric fistula: guiding principles for clinical management and programme development, a new WHO guideline. Int J Gynecol Obstet.

[18] Kelly SE, Bourgeault I, Dingwall R (2010). Qualitative interviewing techniques and styles. The SAGE handbook of qualitative methods in health research.

[19] Kallio H, Pietilä AM, Johnson M, Kangasniemi M (2016). Systematic methodological review: developing a framework for a qualitative semi-structured interview guide. J Adv Nurs.

[20] Clarke V, Braun V (2013). Teaching thematic analysis: overcoming challenges and developing strategies for effective learning. Psychologist.

[21] Braun V, Clarke V, Hayfield N, Terry G (2019). Thematic analysis. Handbook of Research Methods in Health Social Sciences.

[22] Muleta M (2006). Obstetric fistula in developing countries: a review article. J Obstet Gynaecol Can.

[23] Kavai MM, Chepchirchir A, Kayugira R (2010). Women’s knowledge of vesico vaginal fistula in Kenya. Afr J Midwifery Womens Health.

[24] Khisa AM, Nyamongo IK (2011). What factors contribute to obstetric fistulae formation in rural Kenya. Afr J Midwifery Womens Health.

[25] John TW, Mkoka DA, Frumence G, Goicolea I (2018). An account for barriers and strategies in fulfilling women's right to quality maternal health care: a qualitative study from rural Tanzania. BMC Pregnancy Childbirth.

[26] Woldeamanuel SA (2012). Factors contributing to the delay in seeking treatment for women with obstetric fistula in Ethiopia.

[27] Tinuola F, Okau A (2009). Perceived causes of obstetric fistula: data from women of reproductive age in Nigeria. Eur J Soc Sci.

[28] Mselle LT, Kohi TW (2016). Healthcare access and quality of birth care: narratives of women living with obstetric fistula in rural Tanzania. Reprod Health.

[29] Watt MH, Wilson SM, Joseph M, Masenga G, MacFarlane JC, Oneko O (2014). Religious coping among women with obstetric fistula in Tanzania. Global Public Health.

