# Supplementary material for: Reasons for delay in seeking treatment among women with obstetric fistula in Tanzania: a qualitative study
Source: BMC Womens Health. 2019 Jul 10;19:93. doi: 10.1186/s12905-019-0799-x (PMC6617583; doi:10.1186/s12905-019-0799-x)
Supplement: Supplementary file 1 — Patients selection process flow chart. (DOCX 26 kb) [file 12905_2019_799_MOESM1_ESM.docx]

**Additional file 1: Patients selection process flow chart**
